# The Effect of Dose Adjustments in a Subsequent Cycle of Women With Suboptimal Response Following Conventional Ovarian Stimulation

**DOI:** 10.3389/fendo.2018.00361

**Published:** 2018-07-23

**Authors:** Panagiotis Drakopoulos, Samuel Santos-Ribeiro, Ernesto Bosch, Juan Garcia-Velasco, Christophe Blockeel, Alessia Romito, Herman Tournaye, Nikolaos P. Polyzos

**Affiliations:** ^1^Department of Surgical and Clinical Science, Faculty of Medicine and Pharmacy, Vrije Universiteit Brussel, Brussels, Belgium; ^2^Center for Reproductive Medicine, Universitair Ziekenhuis Brussel, Brussels, Belgium; ^3^Department of Reproductive Medicine, University of Liège, Liège, Belgium; ^4^Department of Obstetrics, Gynaecology and Reproductive Medicine, Santa Maria University Hospital, Lisbon, Portugal; ^5^Instituto Valenciano de Infertilidad (IVI-RMA), Valencia, Spain; ^6^Instituto Valenciano de Infertilidad (IVI-RMA), Madrid, Spain; ^7^Department of Obstetrics and Gynecology, Rey Juan Carlos University, Madrid, Spain; ^8^Department of Obstetrics and Gynaecology, University of Zagreb-School of Medicine, Zagreb, Croatia; ^9^Department of Reproductive Medicine, Dexeus University Hospital, Barcelona, Spain; ^10^Department of Clinical Medicine, Faculty of Health, Aarhus University, Aarhus, Denmark

**Keywords:** oocytes, ovarian response, suboptimal responders, number of oocytes, dose adjustments

## Abstract

Several infertile patients, who may even represent around 40% of the infertile cohort, may respond “suboptimally” (4–9 oocytes retrieved) following IVF, despite being predicted as normal responders. The aim of our longitudinal study was to evaluate the ovarian response of suboptimal responders in terms of the number of oocytes retrieved, following their second IVF cycle, evaluating exclusively patients who had the same stimulation protocol and used the same or higher initial dose of the same type of gonadotropin compared to their previous failed IVF attempt. Overall, our analysis included 160 patients treated with a fixed antagonist protocol in their second cycle with the same [53 (33.1%)] or higher [107 (66.9%)] starting dose of rFSH. The number of oocytes retrieved was significantly higher in the second IVF cycle [6 (5–8) vs. 9 (6–12), *p* < 0.001]. According to our results, a dose increment of rFSH remained the only significant predictor of the number of oocytes retrieved in the subsequent IVF cycle (coefficient 0.02, *p*-value = 0.007) after conducting GEE multivariate regression, while adjusting for relevant confounders. A regression coefficient of 0.02 for the starting dose implies that an increase of 50 IU of the initial rFSH dose would lead to 1 more oocyte.

## Introduction

The number of oocytes retrieved following ovarian stimulation is considered to be a strong surrogate marker for the reproductive outcome. Since the early days of *in-vitro* fertilization (IVF), ovarian stimulation has been applied to compensate for inefficiencies in the IVF procedure by aiming to increase the oocyte yield. While there is scientific evidence to justify the categorization of women as poor responders (≤3 oocytes) or excessive responders (>15 oocytes) based on a uniform prognosis, categorization of patients as normal responders is often based on the exclusion of the aforementioned categories (1).

The homogeneity of this “normal” group has been recently debated, given that patients with 4–9 retrieved oocytes may have substantial different clinical prognosis in comparison to women with a 10–15 oocyte yield (2). This implies that several patients, who may even represent around 40% of the infertile cohort (3), may respond “suboptimally” following ovarian stimulation, despite being predicted as normal responders based on their ovarian reserve markers (4).

Although several explanations may be given for the nature of suboptimal response, the main dilemma is which treatment modality should be implemented in order to increase the number of oocytes in a subsequent IVF cycle (5). In this context, the adjustment of the gonadotropins' dose in a following cycle represents one of the most common treatment measures used in clinical practice. However, in order to be able to evaluate this approach, the naturally existing individual variability in ovarian response between consecutive cycles should be taken into consideration and for such an assessment, repetitive cycles should be evaluated, which would ideally be performed under the same conditions.

Therefore, the aim of our study was to evaluate the ovarian response of suboptimal responders in term of number of oocytes retrieved, following their second IVF cycle, evaluating exclusively patients who had the same stimulation protocol and used the same or higher initial dose of the same type of gonadotropin compared to their previous failed attempt. Allowing each patient to serve as her own control could assess inter-patient variability and would provide potential implications for the management of this difficult group of patients.

## Materials and methods

This retrospective study included all consecutive women attending the Centre for Reproductive Medicine (CRG) of the University Hospital of Brussels in Belgium from January 2009 to December 2014. The study was approved by the institutional review board of our hospital (B.U.N. 143201733041).

### Patients' eligibility criteria

Eligible patients were considered to be all consecutive infertile women less than 40 years undergoing their 2nd ovarian stimulation cycle in a fixed gonadotropin-releasing hormone (GnRH) antagonist protocol with daily recombinant FSH (rFSH) and who had demonstrated suboptimal response (4–9 oocytes retrieved) following their 1st IVF cycle with 150 IU of rFSH in an antagonist setting in a time interval less than 12 months.

All the included patients were supposed to be normal responders based on their ovarian reserve markers [anti-müllerian hormone (AMH) and antral follicle count (AFC)] and may have used the same initial dose or ≥25 IU increase in rFSH in their 2nd IVF cycle, based on clinicians' discretion.

Patients were excluded from the study if they did not proceed to a 2nd IVF attempt, if they had undergone ovarian stimulation with a GnRH agonist protocol, if they had been stimulated with urinary gonadotropins or if the time interval between the two oocyte retrievals was longer than 12 months.

**Figure 1:**
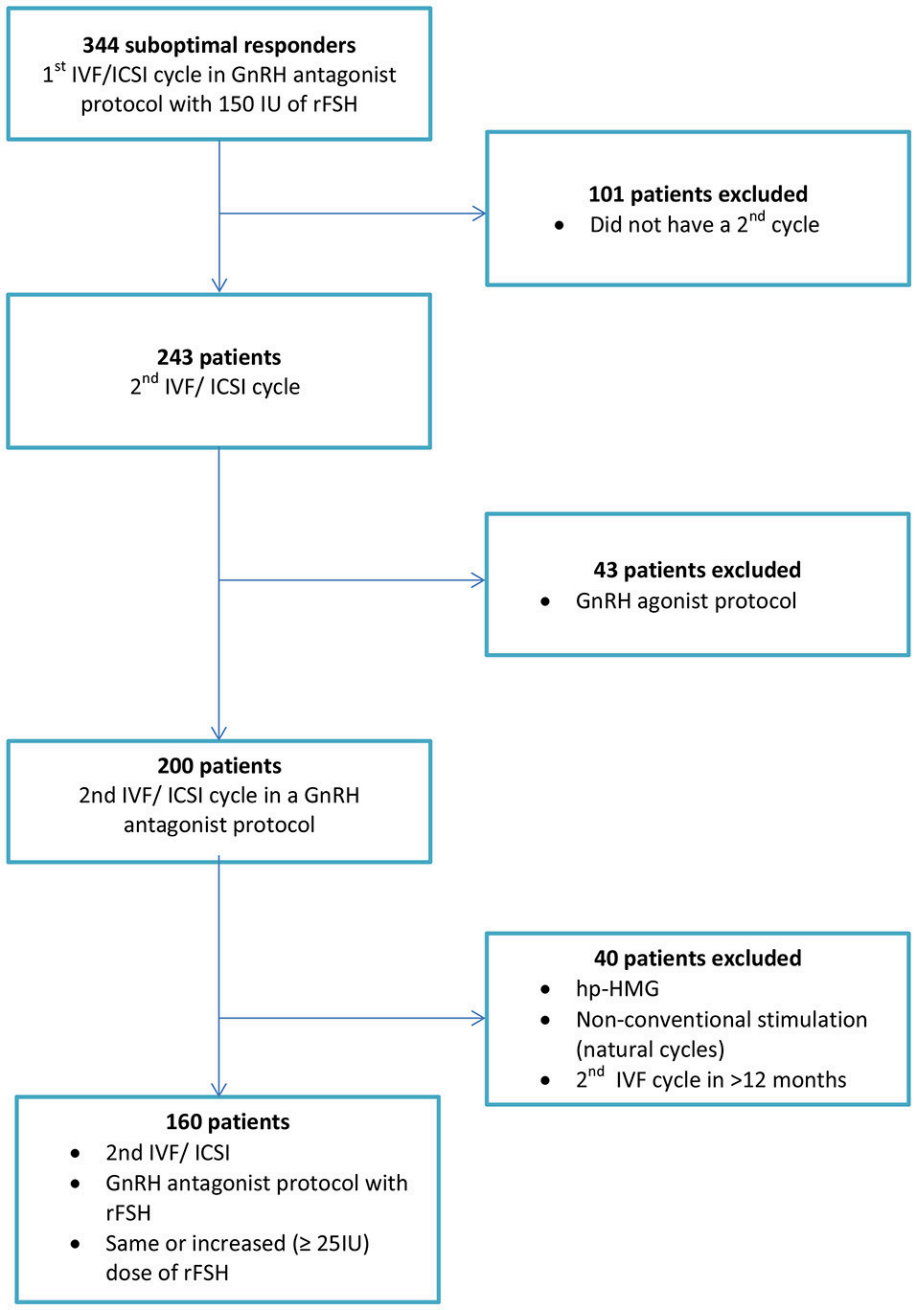
Flowchart of the included population.

In addition, we excluded women who were planned to undergo ovarian stimulation for pre-implantation genetic diagnosis or screening, oocyte donation, and social or medical freezing of oocytes (Figure 1).

### Treatment protocol

Patients received daily injections of rFSH starting on day 2 or 3 of their menstrual cycle, followed by a daily dose of 0.25 mg of GnRH antagonist in fixed protocol starting 6 days later, as described elsewhere (2). Women did not receive any type of priming before starting IVF. Cycle monitoring was performed through serum estradiol (E2), progesterone and luteinizing hormone (LH) assessments, and serial transvaginal ultrasound examinations. Dose adjustments were not allowed during ovarian stimulation.

Ovulation triggering was performed with the administration of human (10.000IU) or recombinant (250 μcg) chorionic gonadotropin (hCG) as soon as three follicles of 17 mm diameter were observed. Oocyte retrieval took place 36 h later.

### Main outcome measures

The primary objective was to determine the variation between the two treatment cycles following gonadotropin dose adjustment of the initial stimulation dose, in term of number of oocytes retrieved and to investigate whether the changes in the ovarian response per cycle could be explained by several predictors, in suboptimal responders. Secondary endpoint was the total number of good quality Day 3 embryos between cycles. EQ was classified similar to what is described in a previous study performed by De Munck et al. (6), with a minor update in the classification (good quality embryos included up to <50% fragmentation).

### Statistical methods

Continuous data are presented as the mean value ± standard deviation (SD) and median with interquartile range (IQR). Categorical data are described by number of cases, including numerator/denominator and percentages.

Differences in continuous variables (including our primary endpoint: number of oocytes) between patients' 2nd IVF cycle and their preceding cycle are calculated via dependent-sample *t*-tests or Wilcoxon signed-rank tests, as appropriate. Categorical variables are analyzed via chi-square with Fisher exact test, as appropriate.

We also performed regression models with estimation by generalized estimating equations (GEE) to assess the effect of dose adjustments in the number of oocytes and number of good quality Day 3 embryos, after accounting for several confounders. The candidate confounders were age, BMI, cause of infertility and AFC. GEE were used to account for the within subject correlation in outcomes for repeated treatments. Results are presented with adjusted odds ratios (ORs) and 95% confidence intervals (CIs). All statistical tests used a two-tailed α of 0.05. All analyses performed using STATA 13.0.

## Results

### Patient characteristics according to number of oocytes retrieved in the two IVF cycles

Overall, our longitudinal analysis included 160 suboptimal responders treated with a fixed GnRH antagonist in their second IVF cycle with the same [53 (33.1%)] or higher [107 (66.9%)] starting dose of rFSH.

The patients' baseline characteristics are presented in Table 1. Comparisons between the two IVF cycles revealed significant differences in the initial and total rFSH dose (Table 2). However, the duration of stimulation was comparable between the two cycles.

**Table 1:** Baseline characteristics of suboptimal responders.

**AGE (YEARS)**	
Mean (SD)	32 (4.5)
Median (IQR)	32 (29–35)
**BMI (kg/m^2^)**	
Mean (SD)	23.5 (4.4)
Median (IQR)	22.4 (20–26)
**INFERTILITY CAUSE, n(%)**	
Male	87 (54.4)
Endometriosis	2 (1.25)
PCOS	7 (4.4)
Ovulatory	7 (4.4)
Tubal	10 (6.25)
Unexplained	47 (29.4)
**FSH**	
Mean (SD)	7.4 (2.5)
Median (IQR)	7 (6–8.8)
**AMH**	
Mean (SD)	3 (2)
Median (IQR)	2.8 (1.9–4)
**AFC**	
Mean (SD)	15 (7.8)
Median (IQR)	14 (10–18)
**TIME INTERVAL BETWEEN ORs (DAYS)**	
Mean (SD)	132 (68)
Median (IQR)	115 (82–174)

*AFC, antral follicle count*.

*OR, oocyte retrieval*.

**Table 2:** Ovarian stimulation outcomes.

**Same dose (150 IU) of rFSH in the 2nd cycle (*n* = 53)**	**1st IVF/ICSI cycle**	**2nd IVF/ICSI cycle**
**Increased dose (226525IU) of rFSH in the 2nd cycle (*n* = 107)**	***n* = 160**	***n* = 160**
INITIAL DOSE (IU)^***^		
Mean (SD)	150	194(42)
Median (IQR)	150	200(150–200)
STIMULATION UNITS (TOTAL IU)^***^		
Mean (SD)	1,434(493.7)	1,775(589)
Median (IQR)	1,350(1,200–1,500)	1,668(1,350–2,000)
DURATION OF STIMULATION (DAYS)^*^		
Mean (SD)	9.6 (2.4)	9.5 (1.8)
Median (IQR)	9 (8–11)	9 (8–11)
NUMBER OF OOCYTES^***^^§^		
Mean (SD)	6.5(1.6)	9.3(4.8)
Median (IQR)	6(5–8)	9(6–12)
NUMBER OF GOOD QUALITY DAY3 EMBRYOS^***^		
Mean (SD)	2.9(1.6)	4(3)
Median (IQR)	3(2–4)	4(2–5)

Wilcoxon signed-rank test significant at ^***^ < 0.01, not significant at ^*^>0.5.

*^§^For patients having the same dose (150IU) in the 2nd IVF cycle, the mean difference in the number of oocytes between cycles was 2.1 (p < 0.05), while for patients having a higher dose of rFSH, the mean difference in the number of oocytes between cycles was 3.2 (p < 0.05)*.

The number of oocytes retrieved and good quality embryos were significantly higher in the second IVF cycle [6 (5–8) vs. 9 (6–12) and 3(2–4) vs. 4(2–5), respectively, *p* < 0.001].

### Generalized estimating equation (GEE) regression analysis for number of oocytes retrieved and good quality embryos in the 2nd IVF cycle

**Figure 2:**
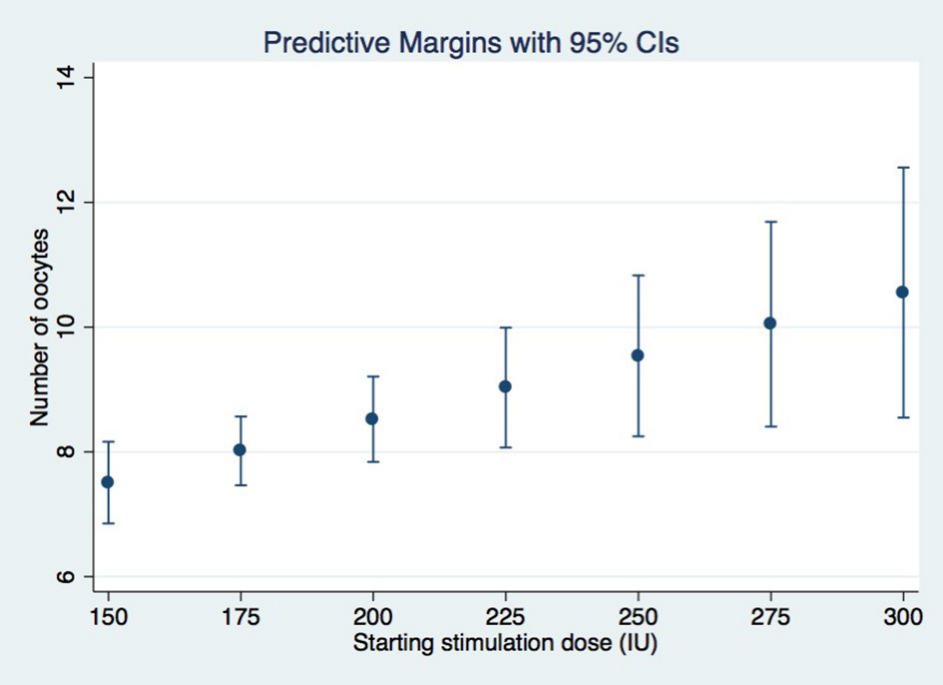
Mean number of oocytes according to the dose of rFSH.

A dose increment of rFSH remained the only significant predictor of the number of oocytes retrieved in the subsequent IVF cycle (coefficient 0.02, *p*-value = 0.007) of suboptimal responders after conducting GEE multivariate regression, while adjusting for relevant confounders (Table 3). Age, BMI, cause of infertility and AFC were not significantly associated with the oocyte yield of the 2nd IVF cycle. Figure 2 represents the mean number of oocytes according to the dose of rFSH given, adjusting for the clustering among patients. Similarly, the dose increment had a positive (although non-significant) effect in the number of good quality Day 3 embryos (Supplementary Table I).

**Table 3:** Generalized estimating equation (GEE) regression analysis for number of oocytes retrieved in the 2nd IVF cycle.

**Number of oocytes in the 2nd IVF cycle**	**Coefficient**	**95% CI**	***P*-value**
Dose increment	0.02	0.005 to 0.04	0.009
Age	−0.05	−0.18 to 0.07	0.4
BMI	−0.09	−0.22 to 0.05	0.19
**CAUSE OF INFERTILITY**			
PCOS	–	–	
Tubal	1.5	-2.2 to 5.3	
Endometriosis	−1.9	−8.6 to 4.8	0.77
Male	1.03	−2.1 to 4.23	
Ovarian	0.7	−3.6 to 5.06	
Unexplained	1.5	1.79 to 4.7	
AFC	0.07	−0.008 to 0.16	0.08

## Discussion

To the best of our knowledge, this is the first study to examine the effect of dose adjustments in a subsequent IVF cycle, using the same stimulation protocol and type of gonadotropin in suboptimal responders. Our study demonstrated that an increase in the dose of rFSH in women with a previous suboptimal response may significantly increase the number of oocytes retrieved in the following IVF cycle. Based on our results, a regression coefficient of 0.02 for the starting stimulation dose implies that an increase of 50 IU of the initial rFSH dose would lead to 1 more oocyte. This average increase of one oocyte by 50 IU increment of rFSH dose may be clinically relevant for women who fail an initial IVF attempt, given the delivery rate of 5% per oocyte with IVF (3, 7, 8). The increase in the oocyte yield was also translated to a higher number of good quality cleavage stage embryos; albeit the positive effect of dose increase was not statistically significant after adjustment for confounders.

Although many theories have been investigated for the nature of suboptimal response, it seems that a decreased sensitivity- or insensitivity of follicles to FSH (9) may be the most likely explanation. In fact, there is evidence that genetic variations of FSH receptor (FSHR) influence serum FSH levels and the physiological responsiveness of the target organ to FSH stimulation (10). If we further consider that no significant associations between FSHR polymorphisms and ovarian reserve markers have been found (11), women with suboptimal response may belong to this group and, therefore, require higher gonadotropin doses, contrary to their predicted response based on ovarian reserve markers.

Our study is one of the largest evaluating the variability of ovarian response in subsequent ovarian stimulation cycles, given that each patient served as her own control. Although several previous studies have investigated the ovarian response by comparing outcomes of subsequent cycles in a period of several years (12, 13), firm conclusions about the effect of ovarian stimulation can only be drawn if repetitive cycles are performed ideally within a short time frame, using the same stimulation strategy (e.g., type of gonadotropin, GnRH analog protocol, and decisions on patient management). In this regard, evidence derived from oocyte donation cycles has shown that the ovarian response is not altered in case a subsequent IVF cycle is started in a short period after the first attempt (14, 15). However, these studies evaluated the effect of stimulation on reproductive outcomes without taking into account the ovarian stimulation regimen, which is the most important parameter for decision-making. Our study differs significantly from those available in the literature, since all patients were infertile, had the same stimulation protocol with rFSH and used the same or higher initial dose of stimulation after their first failed suboptimal response. Our results correlate with a previous retrospective cohort study demonstrating that an increase in the average daily dose of gonadotropins was the only variable significantly associated with a higher oocyte yield in women with normal ovarian reserve, undergoing two IVF cycles (16).

Another point of discussion is that according to our results, the variability in ovarian response was, however, not strongly linked to individual patient demographics or baseline predictors. This is in agreement with two previous studies showing that neither basal FSH nor AFC could significantly predict transition in ovarian response following consecutive IVF cycles (16, 17).

One of the major strengths of our retrospective longitudinal study is that we included a large homogeneous group of women who had the same stimulation protocol and the same type of gonadotropin in their second treatment cycle, performed in a short time interval. The rationale for such a study design was to take into account the individual variability by repeated measurements, eliminate potential confounders and be able to evaluate the dose adjustment “*per se*.” Our study design reflects evidence based clinical practice given that all women had their first stimulation cycle with 150 IU of rFSH, based on the fact that they were predicted as normal responders (18).

However, caution is needed owing to limitations that do exist and need to be highlighted. First of all, the retrospective study design is per definition associated with inherent biases that may affect our results. Although the relatively greater oocytes yield with dose increment, this could represent a regression to the mean (17). Secondly, we excluded women who had a second IVF cycle in more than 12 months after their first egg retrieval. Nevertheless, our strategy was to decrease as much as possible the confounding effects, especially of age, by choosing a time interval in which the predictive ability of ovarian reserve markers has been shown to be the same (19). Thirdly, the adjustment in the starting stimulation dose of the subsequent cycle was based on the clinicians' discretion, with approximately one third of the patients keeping the same rFSH dose and two thirds having an increase in their initial dose. However, such an approach reflects current clinical practice and the regression analysis allowed to adjust for confounders and methodologically corroborate that the common strategy of dose increase in case of suboptimal response could be beneficial. Fourthly, patients were categorized as normal responders based on ovarian reserve biomarkers. Even if comparisons of AFC and AMH levels have generally yielded similar predictive value for ovarian response in 3 meta-analyses (20–22), limitations do exist. The major disadvantages of AFC are the sonographer dependent variability and problems related to technical aspects of ultrasound equipment (23), while the main limitations of the AMH test relate to assay variability and lack of standardized international assay (24). Finally, although the number of oocytes was found to increase with a higher starting dose, our design cannot allow evaluating the effect on fresh and cumulative live birth rates. The fact that the stimulation initial dose increase was related to a significant higher number of oocytes, but not good quality embryos, may be due to a Type 2 error.

In conclusion, after a failed cycle with suboptimal response, physicians review the cycle and often change stimulation protocol or gonadotropin dosing in an attempt to improve the outcome. By using a robust methodological approach, we answered one of the main queries, namely that an increase in the initial stimulation dose may significantly increase the oocyte yield in suboptimal responders. Our study could generate a hypothesis for a prospective randomized trial, in which suboptimal responders would be allocated to two different groups:one group with the same starting dose and a second group with a higher starting dose. The primary endpoint could be the oocyte yield and the follicular output ratio (FORT), as a qualitative marker of ovarian response (25). If we further consider that several suboptimal responders may have a variant of the β subunit of luteinizing hormone (LH) (v-LH) affecting FSH sensitivity (26, 27), the co-administration or rLH may also represent a valid option (28). However, further studies are urgently needed, in order to evaluate these promising concepts.

## Author contributions

PD and NP contributed to the concept and the design of the study. PD was responsible for the data management, interpretation of the results, statistical analysis and he drafted the manuscript. All authors contributed to the interpretation of the results and editing of the manuscript. All authors approved the final version of the manuscript.

### Conflict of interest statement

The authors declare that the research was conducted in the absence of any commercial or financial relationships that could be construed as a potential conflict of interest.

## References

[1] Polyzos NP Sunkara SK. Sub-optimal responders following controlled ovarian stimulation: an overlooked group? Hum Reprod. (2015) 30:2005–8. 10.1093/humrep/dev149 26202582

[2] Drakopoulos P Blockeel C Stoop D Camus M de Vos M Tournaye H. Conventional ovarian stimulation and single embryo transfer for IVF/ICSI. How many oocytes do we need to maximize cumulative live birth rates after utilization of all fresh and frozen embryos? Hum Reprod. (2016) 31:370–6. 10.1093/humrep/dev316 26724797

[3] Sunkara SK Rittenberg V Raine-Fenning N Bhattacharya S Zamora J Coomarasamy A. Association between the number of eggs and live birth in IVF treatment: an analysis of 400 135 treatment cycles. Hum Reprod. (2011) 26:1768–74. 10.1093/humrep/der106 21558332

[4] Poseidon G Alviggi C Andersen CY Buehler K Conforti A De Placido G. A new more detailed stratification of low responders to ovarian stimulation: from a poor ovarian response to a low prognosis concept. Fertil Steril. (2016) 105:1452–3. 10.1016/j.fertnstert.2016.02.005 26921622

[5] Rombauts L. Is there a recommended maximum starting dose of FSH in IVF? J Assist Reprod Genet. (2007) 24:343–9. 10.1007/s10815-007-9134-9 17574524 PMC3454944

[6] De Munck N Santos-Ribeiro S Mateizel I Verheyen G. Reduced blastocyst formation in reduced culture volume. J Assist Reprod Genet. (2015) 32:1365–70. 10.1007/s10815-015-0541-z 26292800 PMC4595393

[7] Patrizio P Sakkas D. From oocyte to baby: a clinical evaluation of the biological efficiency of *in vitro* fertilization. Fertil Steril. (2009) 91:1061–6. 10.1016/j.fertnstert.2008.01.003 18325517

[8] Martin JR Bromer JG Sakkas D Patrizio P. Live babies born per oocyte retrieved in a subpopulation of oocyte donors with repetitive reproductive success. Fertil Steril. (2010) 94:2064–8. 10.1016/j.fertnstert.2010.02.004 20303483

[9] Simoni M Nieschlag E Gromoll J. Isoforms and single nucleotide polymorphisms of the FSH receptor gene: implications for human reproduction. Hum Reprod Update (2002) 8:413–21. 10.1093/humupd/8.5.413 12398222

[10] Perez Mayorga M Gromoll J Behre HM Gassner C Nieschlag E Simoni M. Ovarian response to follicle-stimulating hormone (FSH) stimulation depends on the FSH receptor genotype. J Clin Endocrinol Metab. (2000) 85:3365–9. 10.1210/jcem.85.9.6789 10999835

[11] Mohiyiddeen L Newman WG McBurney H Mulugeta B Roberts SA Nardo LG. Follicle-stimulating hormone receptor gene polymorphisms are not associated with ovarian reserve markers. Fertil Steril (2012) 97:677–81. 10.1016/j.fertnstert.2011.12.040 22265040

[12] Hoveyda F Engmann L Steele J Lopez Bernal A Barlow DH. Ovarian response in three consecutive in vitro fertilization cycles. Fertil Steril. (2002) 77:706–10. 10.1016/S0015-0282(01)03237-X 11937120

[13] Doldi N Persico P De Santis L Rabellotti E Papaleo E Ferrari A. Consecutive cycles in *in vitro* fertilization–embryo transfer. Gynecol Endocrinol. (2005) 20:132–6. 10.1080/09513590400021094 16019351

[14] Caligara C Navarro J Vargas G Simon C Pellicer A Remohi J. The effect of repeated controlled ovarian stimulation in donors. Hum Reprod. (2001) 16:2320–3. 10.1093/humrep/16.11.2320 11679512

[15] Jain A Robins JC Williams DB Thomas MA. The effect of multiple cycles in oocyte donors. Am J Obstet Gynecol. (2005) 192:1382–4. 10.1016/j.ajog.2004.12.038 15902115

[16] Eppsteiner EE Sparks AE Liu D Van Voorhis BJ. Change in oocyte yield in repeated *in vitro* fertilization cycles: effect of ovarian reserve. Fertil Steril. (2014) 101:399–402. 10.1016/j.fertnstert.2013.10.049 24331835

[17] Rombauts L Lambalk CB Schultze-Mosgau A van Kuijk J Verweij P Gates D. Intercycle variability of the ovarian response in patients undergoing repeated stimulation with corifollitropin alfa in a gonadotropin-releasing hormone antagonist protocol. Fertil Steril. (2015) 104:884–890 e882. 10.1016/j.fertnstert.2015.06.027 26187300

[18] Sterrenburg MD Veltman-Verhulst SM Eijkemans MJ Hughes EG Macklon NS Broekmans FJ. Clinical outcomes in relation to the daily dose of recombinant follicle-stimulating hormone for ovarian stimulation in *in vitro* fertilization in presumed normal responders younger than 39 years: a meta-analysis. Hum Reprod Update (2011) 17:184–96. 10.1093/humupd/dmq041 20843965

[19] Polyzos NP Nelson SM Stoop D Nwoye M Humaidan P Anckaert E. Does the time interval between antimullerian hormone serum sampling and initiation of ovarian stimulation affect its predictive ability in *in vitro* fertilization-intracytoplasmic sperm injection cycles with a gonadotropin-releasing hormone antagonist? A retrospective single-center study. Fertil Steril. (2013) 100:438–44. 10.1016/j.fertnstert.2013.03.031 23602319

[20] Broekmans FJ Kwee J Hendriks DJ Mol BW Lambalk CB. A systematic review of tests predicting ovarian reserve and IVF outcome. Hum Reprod Update (2006) 12:685–718. 10.1093/humupd/dml034 16891297

[21] Broer SL Mol BW Hendriks D Broekmans FJ. The role of antimullerian hormone in prediction of outcome after IVF: comparison with the antral follicle count. Fertil Steril. (2009) 91:705–714. 10.1016/j.fertnstert.2007.12.013 18321493

[22] Broer SL van Disseldorp J Broeze KA Dolleman M Opmeer BC Bossuyt P. Added value of ovarian reserve testing on patient characteristics in the prediction of ovarian response and ongoing pregnancy: an individual patient data approach. Hum Reprod Update (2013) 19:26–36. 10.1093/humupd/dms041 23188168

[23] Broekmans FJ de Ziegler D Howles CM Gougeon A Trew G Olivennes F. The antral follicle count: practical recommendations for better standardization. Fertil Steril. (2010) 94:1044–51. 10.1016/j.fertnstert.2009.04.040 19589513

[24] Broer SL Broekmans FJ Laven JS Fauser BC. Anti-Mullerian hormone: ovarian reserve testing and its potential clinical implications. Hum Reprod Update (2014) 20:688–701. 10.1093/humupd/dmu020 24821925

[25] Genro VK Grynberg M Scheffer JB Roux I Frydman R Fanchin R. Serum anti-Mullerian hormone levels are negatively related to Follicular Output RaTe (FORT) in normo-cycling women undergoing controlled ovarian hyperstimulation. Hum Reprod. (2011) 26:671–7. 10.1093/humrep/deq361 21177311

[26] Alviggi C Clarizia R Pettersson K Mollo A Humaidan P Strina I. Suboptimal response to GnRHa long protocol is associated with a common LH polymorphism. Reprod Biomed Online (2009) 18:9–14. 10.1016/S1472-6483(10)60418-X 19146763

[27] Alviggi C Pettersson K Longobardi S Andersen CY Conforti A De Rosa P. A common polymorphic allele of the LH beta-subunit gene is associated with higher exogenous FSH consumption during controlled ovarian stimulation for assisted reproductive technology. Reprod Biol Endocrinol. (2013) 11:51. 10.1186/1477-7827-11-51 23725475 PMC3694457

[28] Papaleo E Vanni VS Vigano P La Marca A Pagliardini L Vitrano R. Recombinant LH administration in subsequent cycle after “unexpected” poor response to recombinant FSH monotherapy. Gynecol Endocrinol. (2014) 30:813–6. 10.3109/09513590.2014.932342 24968088

