# Supplementary material for: The Effect of Dose Adjustments in a Subsequent Cycle of Women With Suboptimal Response Following Conventional Ovarian Stimulation
Source: Front Endocrinol (Lausanne). 2018 Jul 23;9:361. doi: 10.3389/fendo.2018.00361 (PMC6064928; doi:10.3389/fendo.2018.00361)
Supplement: Supplementary file 1 [file Table_1.docx]

| **Number of good quality cleavage stage embryos in the 2^nd^ IVF cycle** | **Coefficient** | **P- value** |
| --- | --- | --- |
| Dose increment | 0.018 | 0.7 |
| Age | -0.04 | 0.3 |
| BMI | -0.08 | 0.08 |
| Cause of infertility  PCOS  Tubal  Endometriosis  Male  Ovarian  Unexplained | -  0.6  -1.4  0.3  1.6  1.3 | 0.2 |
| AFC | 0.05 | 0.3 |

**Supplementary Table 1:** Generalized estimating equation (GEE) regression analysis for number of good quality Day 3 embryos in the 2^nd^ IVF cycle
